# Vitamin A-Deficient Diet Accelerated Atherogenesis in Apolipoprotein E^−/−^ Mice and Dietary *β*-Carotene Prevents This Consequence

**DOI:** 10.1155/2015/758723

**Published:** 2015-02-23

**Authors:** Noa Zolberg Relevy, Dror Harats, Ayelet Harari, Ami Ben-Amotz, Rafael Bitzur, Ralph Rühl, Aviv Shaish

**Affiliations:** ^1^The Bert W. Strassburger Lipid Center, Sheba Medical Center, 5265601 Tel-Hashomer, Israel; ^2^Sackler Faculty of Medicine, Tel-Aviv University, Israel; ^3^N.B.T., Eilat, Israel; ^4^Department of Biochemistry and Molecular Biology, University of Debrecen, Debrecen, Hungary

## Abstract

Vitamin A is involved in regulation of glucose concentrations, lipid metabolism, and inflammation, which are major risk factors for atherogenesis. However, the effect of vitamin A deficiency on atherogenesis has not been investigated. Therefore, the objective of the current study was to examine whether vitamin A deficiency accelerates atherogenesis in apolipoprotein E-deficient mice (apoE^−/−^). ApoE^−/−^ mice were allocated into the following groups: control, fed vitamin A-containing chow diet; BC, fed chow diet fortified with *Dunaliella* powder containing *β*c isomers; VAD, fed vitamin A-deficient diet; and VAD-BC group, fed vitamin A-deficient diet fortified with a *Dunaliella* powder. Following 15 weeks of treatment, liver retinol concentration had decreased significantly in the VAD group to about 30% that of control group. Vitamin A-deficient diet significantly increased both plasma cholesterol concentrations and the atherosclerotic lesion area at the aortic sinus (+61%) compared to the control group. Dietary *β*c fortification inhibited the elevation in plasma cholesterol and retarded atherogenesis in mice fed the vitamin A-deficient diet. The results imply that dietary vitamin A deficiency should be examined as a risk factor for atherosclerosis and that dietary *β*c, as a sole source of retinoids, can compensate for vitamin A deficiency.

## 1. Introduction

Vitamin A is involved in regulation of glucose concentrations, lipid metabolism, and inflammation, which are major risk factors for atherosclerosis development [1]. All-*trans* retinoic acid (RA), the endogenous ligand of the retinoic acid receptors (RARs), inhibited experimental atherosclerosis [2, 3] and increased the process of reverse cholesterol transport from macrophages [4]. In previous studies, we demonstrated that feed fortification with *β*-carotene, a vitamin A precursor, can inhibit atherosclerosis in mouse models [5, 6]. Nonetheless, the effect of dietary vitamin A deficiency on atherogenesis has not been studied.

The dietary sources of vitamin A are retinol and mainly retinyl esters from animal origin or provitamin A carotenoids from plant sources comprising numerous isomers of *β*-carotene [7]. Vitamin A deficiency can lead to night blindness, abnormal embryonic development, and various pathological conditions [8]. The major cause of deficiency is low dietary intake of vitamin A, common in developing countries [9]. Several other conditions, such as inflammatory bowel disease, recurrent pancreatitis, excessive alcohol consumption, iron deficiency, and mutation in the *β*-carotene 15,15′ monooxygenase 1 (BCMO1) gene can also cause vitamin A deficiency [10–13].

Dietary vitamin A (retinol, retinyl esters, and provitamin A carotenoids) is transported in chylomicrons to the liver and extrahepatic tissues following intestinal absorption. It is important to note that 66–75% of vitamin A is transported to the liver and stored in hepatic stellate cells (HSCs), while 25–33% is transported directly to various extrahepatic tissues [14]. The liver secretes retinol bound to retinol-binding protein 4 (RBP4) in order to meet tissue needs [15]. In addition, plasma all-*trans* RA can also contribute to tissue RA pools [16]. Although retinol bound to RBP4 is the main source of extrahepatic tissues, dietary vitamin A can compensate for the absence of RBP4 in both mice and humans [17, 18].

The effect of dietary vitamin A deficiency on plasma lipid profiles has been investigated. In one study, a vitamin A-deficient diet decreased HDL cholesterol and had no effect on non-HDL cholesterol or on triglyceride concentrations in rats fed with this diet for three months [19], while in a second study, a vitamin A-deficient diet decreased both HDL cholesterol and triglycerides [20]. To our knowledge, there is no data regarding the effect of a vitamin A-deficient diet in animals challenged with a high-cholesterol high-fat diet or in animal models for atherosclerosis.

A recent study demonstrated that BCMO1 deficiency in mice prevents the conversion of *β*-carotene to retinoids and leads to impaired lipid metabolism [21]. We previously showed that a diet enriched in 9*-cis*-*β*-carotene (9C*β*C) provided as powder of the alga* Dunaliella bardawil* significantly inhibits atherogenesis in a mouse model [6]. These findings led us to examine whether a vitamin A-deficient diet may accelerate atherogenesis and whether dietary carotenoids as a sole source of vitamin A could compensate for dietary vitamin A deficiency.

## 2. Materials and Methods

### 2.1. Mice

Male, 12-week-old apoE-deficient mice (apoE^−/−^) (C57BL6 background, Jackson Laboratories) were used. Mice were housed in plastic cages on a 12 h light/12 h dark cycle with free access to feed and water. Mice were killed with isoflurane. The Animal Care and Use Committee of Sheba Medical Center, Tel-Hashomer, approved all animal protocols (682/11).

### 2.2. Diet

Two commercial diets were used: a normal, low-fat diet (18% protein, 5% fat; TD2018, Harlan Teklad) containing 15 IU/g vitamin A (4.5 *µ*g retinol/g) and a vitamin A-deficient diet (17.7% protein, 5% fat; TD86143, Harlan Teklad). We used a powder of the alga* Dunaliella bardawil* (a gift from Nikken Sohonsha, Japan) as a source for natural carotenoids. The algal powder contains 6% *β*-carotene (weight/weight), comprised of 50% all*-trans* and 50% 9-*cis* isomers [22]. To prepare the feed, 0.25 L of distilled hot water was mixed with 14 g of gelatin until the solution was clear. Then, 1 kg of powdered feed and* Dunaliella* powder (80 g/kg feed) were thoroughly mixed with the warm gelatin solution. After solidifying, the feed was divided into tablets and stored in the freezer. Feed was replaced every other day to minimize oxidation and degradation of its ingredients.

### 2.3. Study Design

*Experiment 1*. Sixty-eight 12-week-old apoE^−/−^ male mice (weight 25 gr) were allocated into 4 groups, 17 animals per group, according to their body weight, plasma cholesterol, and triglyceride (TG) concentrations. The mice were fed for 15 weeks with the specified diet. The control group was fed normal vitamin A-containing chow diet. The BC group was fed chow diet fortified with the alga* Dunaliella*. The VAD group was fed a vitamin A-deficient diet, and the VAD-BC group was fed a vitamin A-deficient diet fortified with natural carotenoids of the alga* Dunaliella* (80 g powder mixed with 1 kg feed). Mice were killed after 15 weeks.

*Experiment 2*. Fourteen 12-week-old apoE^−/−^ male mice (weight 25 gr) were allocated into two groups with seven animals per group; the control group was fed chow diet and the VAD group was fed a vitamin A-deficient diet. The mice were fed for 15 weeks with the specified diet and were killed after 15 weeks.

### 2.4. Lipid Analysis

We used a colorimetric enzymatic procedure to measure total plasma cholesterol (Chol, Roche/Hitachi, Roche Diagnostics) and TG (triglyceride liquid, Senitinel).

### 2.5. Carotenoid Analysis

BC isomer concentrations in the plasma and in the liver were determined by HPLC according to the method described by Shaish et al. [23].

### 2.6. Retinol Analysis

Retinol extraction from liver and plasma was carried out the same way as *β*-carotene extraction. Briefly, samples (~250 mg liver and 200 *µ*L plasma) were extracted with 2 mL of ethanol containing 10 *µ*M butylated hydroxytoluene. After the addition of 2 mL of hexane and 1 mL of DDW, the samples were mixed and centrifuged for 5 min at 1000 g. The hexane layers of plasma samples were dried under a stream of N_2_. The hexane layers of the liver samples were saponified with 2% KOH in Absolute ETOH for 30 min at 50°C. Then, by adding 2 mL of saline and centrifugation, the hexane layer was removed and dried under a stream of N_2_. Dried samples were suspended in 200 *µ*L methanol, and retinol concentrations were determined by reverse phase HPLC on a Vydac C18 column (201TP-54, 250 × 5 mm, 5 *μ*m particle size; Vydac, Hesperia, CA) with methanol/butanol/water 10 mM ammonium acetate as the mobile phase at a flow rate of 0.8 mL/min. Retinol was detected by monitoring its absorbance at 325 nm and by comparison with the retention times of authentic standards. Results are expressed as nanomoles of retinol per gram wet weight of tissue.

### 2.7. Retinoic Acid Analysis

Concentrations of RAs were determined in mouse plasma by our LC-MS method [24]. In summary, 100 *μ*L plasma was diluted with a threefold volume of isopropanol, vortexed for 10 seconds, put in an ultrasonic bath for 5 minutes, shaken for 6 minutes, and centrifuged at 13,000 rpm in a Heraeus BIOFUGE Fresco at +4°C. After centrifugation, the supernatants were dried in an Eppendorf concentrator 5301 (Eppendorf, Germany) at 30°C. The dried extracts were resuspended with 60 *μ*L of methanol, vortexed, shaken, diluted with 40 *μ*L of 60 mM aqueous ammonium acetate solution, transferred into the autosampler, and subsequently analyzed.

### 2.8. Isolation of Primary Mouse Hepatic Stellate Cells

Mouse livers were collected and washed three times in isotonic saline. Then, HSCs were isolated using Pronase-Collagenase-DNAse lysate followed by OptiPrep (Sigma-Aldrich) density gradient centrifugation as described previously [25].

### 2.9. Glucose Tolerance Test

After 15 weeks, a glucose tolerance test (GTT) was performed on mice that were fasted for 4 hours. Mice were injected ip with 20% D-glucose, 1% of body weight (v/w). Blood glucose concentrations were measured periodically up to 120 minutes.

### 2.10. Assessment of Atherosclerosis in the Aortic Sinus

Atherosclerotic fatty streak lesions were quantified by calculating the lesion areas in the aortic sinus [26].

### 2.11. Fast Protein Liquid Chromatography Analysis of Lipoproteins

Plasma from 5 mice was pooled and serum lipoproteins were separated by size exclusion chromatography using a Superose-6 column (30 cm) on fast protein liquid chromatography [27].

### 2.12. Analysis of Gene Expression by Real-Time PCR

A Nucleospin RNA II kit (Macherey-Nagel) was used for RNA extraction. A high capacity cDNA synthesis kit (Applied Biosystems) was used to perform cDNA synthesis. Quantitative real-time PCR was performed with a 7900HT PCR machine (Applied Biosystems), FastStart Universal Probe Master ROX (Roche), and a FAM-labeled TaqMan primer and probe (Applied Biosystems and Roche) for mouse* Cyp7α* (Mm00484152_mL, Applied Biosystems),* Pparγ* (Mm01184322_mL, Applied Biosystems),* Cyp26a1* (312454, Roche), and* Hmgr* (Mm01282499_mL, Applied Biosystems). We used* Gapdh* (307884, Roche) as a reference gene.

### 2.13. Statistical Analyses

One-way ANOVA was used to compare the treatment effect on atherogenesis, with the post hoc Tukey method used for multiple pairwise comparisons. Repeated measures ANOVA was applied to compare changes in weight gain between the treatment groups over the study period. Significance was considered as *P* < 0.05. Values in the text are means ± SE. All statistics were conducted using SPSS (release 12.0 SPSS Inc.).

## 3. Results

**Figure 1:**
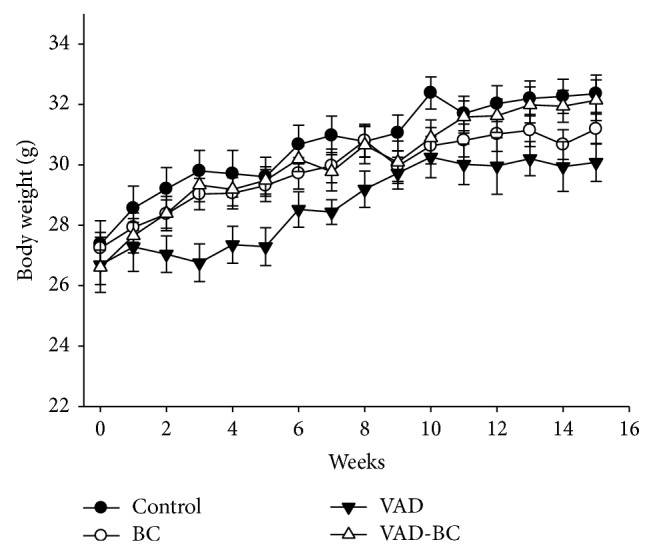
Mouse body weight throughout the study. Mice were weighed every week (Exp. 1). A trend towards lower body weight was noted in the VAD group (*P* = 0.072). Values are means ± SE, *n* = 15-16.* Dunaliella* treated group (BC); vitamin A-deficient diet group (VAD); vitamin A-deficient diet fortified with* Dunaliella* group (VAD-BC).

The aim of the study was to investigate whether a vitamin A-deficient diet accelerates atherogenesis in apoE^−/−^ mice and to examine whether fortification of this diet with a* Dunaliella* powder rich in *β*-carotene isomers, as a sole source of dietary provitamin A, would compensate for dietary vitamin A deficiency. We placed 68 apoE^−/−^ male mice (Exp. 1) for 15 weeks on the following diet regimes: chow diet (control);* Dunaliella* powder-fortified diet, as a natural source for *β*-carotene isomers (BC), vitamin A-deficient diet (VAD), and vitamin A-deficient diet fortified with* Dunaliella* powder (VAD-BC). Vitamin A deficiency did not significantly affect weight gain, although a trend towards lower weight was noted in the VAD group (*P* = 0.072), while weight gain in the BC-fortified groups was similar to the control group (Figure 1).

### 3.1. Vitamin A-Deficient Diet Reduced Liver Retinol Content

**Figure 2:**
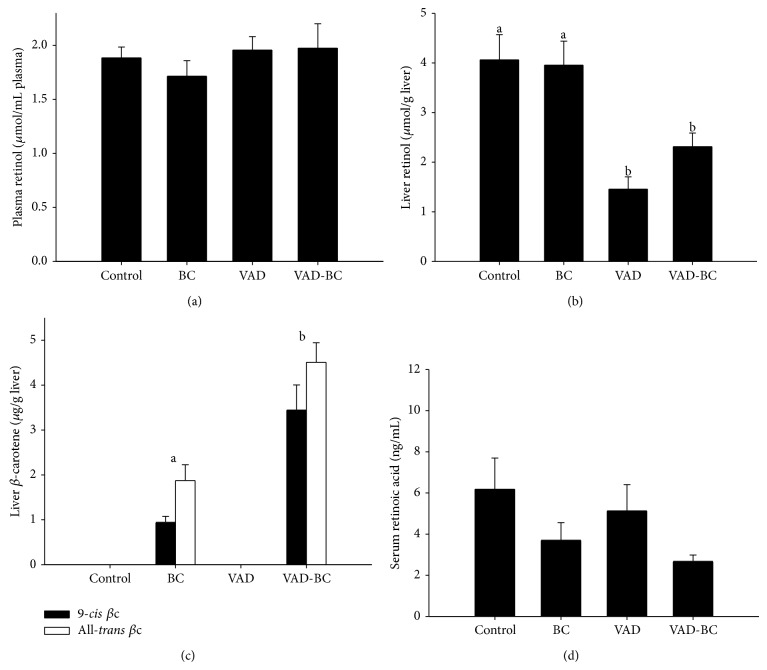
Plasma and liver retinol and *β*-carotene concentrations. Plasma retinol concentrations were similar in all groups (a), liver retinol concentration decreased significantly in VAD group (b), liver *β*c isomer concentrations were significantly higher in VAD-BC group compared to BC group (c), and serum all-*trans *RA concentrations were similar in all groups (d). Retinol, *β*c, and RA were analyzed following 15 weeks of treatments (Exp. 1). Values are means ± SE, *n* = 5–8. ^a, b^Within each graph, means without a common letter differ, *P* < 0.05.* Dunaliella* treated group (BC); vitamin A-deficient diet group (VAD); vitamin A-deficient diet fortified with* Dunaliella* group (VAD-BC); retinoic acid (RA).

Similar to our previous mouse study [6], administration of* Dunaliella* powder rich in *β*-carotene isomers resulted in accumulation of both 9-*cis* and all-*trans*-*β*-carotene isomers in mouse livers. The concentrations of the two isomers were 2-3 times higher in the VAD-BC group compared to the concentrations in the BC group, which also received vitamin A in the form of retinyl ester in the feed (Figure 2(c)). To study whether administration of a vitamin A-deficient diet for 15 weeks led to the vitamin A deficiency in the plasma and liver, we measured retinol concentrations by HPLC in these tissues. Despite the long treatment time of 15 weeks, plasma retinol concentrations were similar in all treatment groups, apparently due to homeostatic regulation (Figure 2(a)). In contrast, liver total retinol concentration was decreased significantly in the VAD group to about 30% that of the control group, indicating that a substantial reservoir of vitamin A still exists (Figure 2(b)). It is important to note that fortification with* Dunaliella* powder rich in *β*-carotene isomers did not restore liver retinol, and only a trend toward higher concentrations was detected compared to the mice treated with a vitamin A-deficient diet.

As most liver vitamin A is stored in hepatic stellate cells, we measured retinol concentrations in isolated stellate cells from control and VAD mice (Exp. 2). As expected, a 50% decline in stellate cell retinol content was detected, 12.9 ng retinol per 10^6^ HSC and 6.7 ng retinol per 10^6^ HSC, respectively.

RA in serum was detected by LC-MS. As RA is one of the vitamin A active derivatives, we assumed that RA concentration would change with respect to diets. In contrast to our assumption that the VAD would reduce and the *β*-carotene would increase serum all-*trans* RA concentrations, all-*trans* RA concentrations were comparable in all examined groups (Figure 2(d)). Moreover, despite the high dose of 9c*β*C in the diet, 9-*cis* RA was not detected in any of the four groups.

### 3.2. Vitamin A-Deficient Diet Increased Plasma Cholesterol Concentrations and Accelerated Atherosclerosis While Dietary *β*-Carotene Reversed These Effects

**Figure 3:**
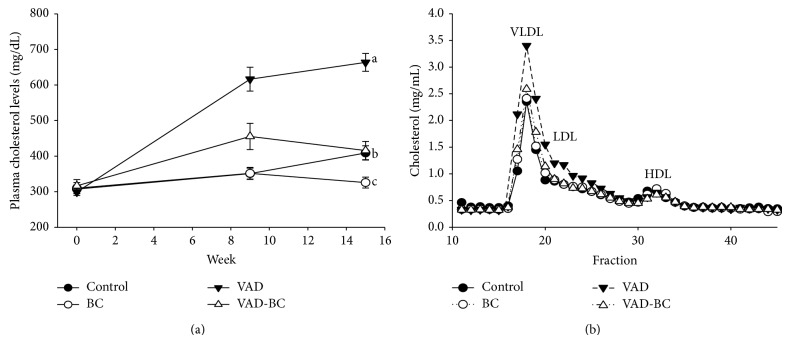
Plasma cholesterol concentrations. Plasma cholesterol concentrations (a) and lipoprotein cholesterol content (b). Mice fed chow or vitamin A-deficient diet with or without *β*C after 15 weeks of treatment. VAD significantly increased plasma cholesterol concentrations due to increased levels of non-HDL cholesterol. Values are means ± SE, *n* = 15-16. ^a, b^Within the graph, means without a common letter differ, *P* < 0.05.* Dunaliella* treated group (BC); vitamin A-deficient diet group (VAD); vitamin A-deficient diet fortified with* Dunaliella* group (VAD-BC).

**Figure 4:**
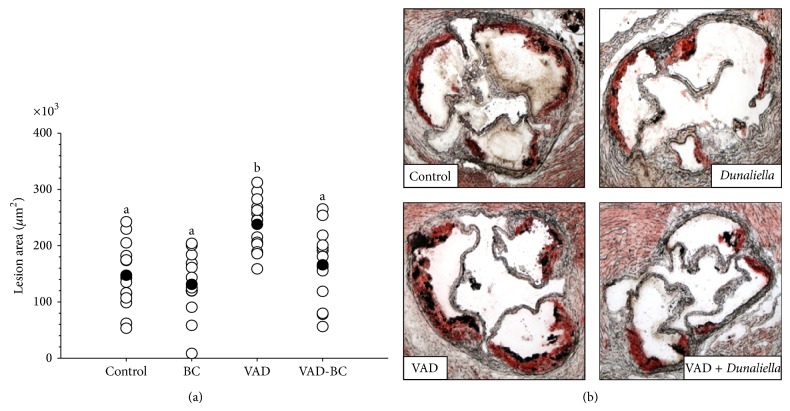
Atherosclerotic lesion area in apoE^−/−^ mice fed chow or vitamin A-deficient diet with or without *β*-carotene. The atherosclerotic lesion area was quantified at the aortic sinus by oil-red O staining of the lipids after 15 weeks of treatment (a). One representative aortic sinus lesion section is shown for each treatment group (magnification ×40). Red color indicates the presence of atherosclerotic lesions (b). The vitamin A-deficient diet significantly increased the atherosclerotic lesion area while *β*-carotene reverts this effect. Values are means ± SE, *n* = 13–15. ^a, b^Within the graph, means without a common letter differ, *P* < 0.05.* Dunaliella* treated group (BC); vitamin A-deficient diet group (VAD); vitamin A-deficient diet fortified with* Dunaliella* group (VAD-BC).

**Figure 5:**
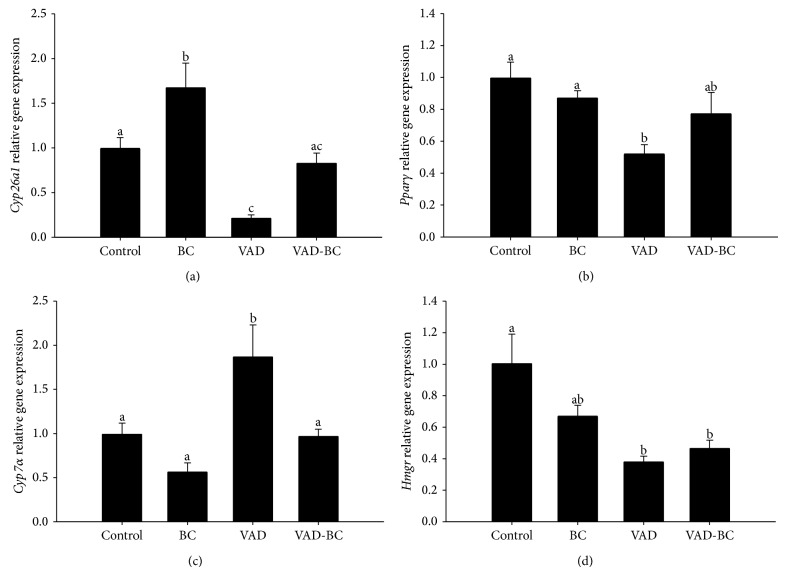
Liver gene expression in apoE^−/−^ mice fed chow or vitamin A-deficient diet with or without *β-*carotene. Liver mRNA levels of the indicated genes were detected by real-time PCR.* Gapdh* was used as a reference gene. Gene expression was detected after 15 weeks of treatment.* Cyp26a1* (a),* Pparγ* (b),* Cyp7α*(c), and* Hmgr *(d). Measured by real-time PCR and normalized to GAPDH. Values are means ± SE, *n* = 8. ^a, b, c^Within each graph, means without a common letter differ, *P* < 0.05.* Dunaliella* treated group (BC); vitamin A-deficient diet group (VAD); vitamin A-deficient diet fortified with* Dunaliella* group (VAD-BC).

Vitamin A-deficient diet significantly increased plasma cholesterol concentrations and FPLC analysis showed that this increase is due to higher concentrations of the atherogenic, non-HDL cholesterol. Dietary *β*-carotene fortification suppressed this elevation, and plasma cholesterol concentrations in the BC and VAD-BC groups were lower throughout the experiments compared to the control or the VAD group, respectively (Figure 3). The atherosclerotic lesion area was quantified at the aortic sinus by oil-red O staining of the lipids. The red color indicates the presence of atherosclerotic lesions (Figure 5(b)). The vitamin A-deficient diet significantly increased (61%) the atherosclerotic lesion area at the aortic sinus compared to the control group (Figure 4). Remarkably, although *β*-carotene did not restore the liver retinol pool, it significantly inhibited atherogenesis in mice fed a vitamin A-deficient diet, and the lesion area was similar to the control group fed the vitamin A-containing diet.

### 3.3. Dietary Vitamin A Deficiency Did Not Induce Insulin Resistance

As retinoids have been shown to impact glucose metabolism, we assessed whether a vitamin A-deficient diet affects glucose concentrations and insulin resistance in mice. The GTT showed that a vitamin A-deficient diet did not induce insulin resistance (data not shown).

### 3.4. The Effect of Vitamin A-Deficient Diet and BC Supplementation on Liver Gene Expression

In an effort to elucidate the mechanisms of cholesterol elevation and accelerated atherogenesis in VAD-treated mice, we measured the liver mRNA levels of several genes related to retinoids and cholesterol metabolism.* Cyp26a1* regulates the cellular concentrations of RA via oxidation and its expression levels are used as a sensitive indicator for tissue RA concentration [28]. Its lower mRNA levels in VAD-treated mice indicate lower RA in the liver of VAD group. It is noteworthy that a diet rich in* Dunaliella* powder rich in *β*-carotene isomers prevented this decrease (Figure 5). Peroxisome proliferator-activated receptor gamma (*Pparγ*) participates in adipocyte differentiation, glucose and insulin homeostasis, and inflammation, and it was suggested that it plays an important role in several diseases, including obesity, diabetes, and atherosclerosis [29]. The mRNA levels of* Pparγ* decreased twofold in VAD-treated mice compared to the control and increased slightly (not significant) in the VAD-BC group.

Cholesterol-7-alpha-hydroxylase (*Cyp7α*) catalyzes the formation of 7-alpha-hydroxycholesterol, which is a rate-limiting step in the synthesis of bile acids from cholesterol. The rate-limited enzyme during cholesterol biosynthesis is 3-hydroxy-3-methyl-glutaryl-CoA reductase (*Hmgr*). In VAD-treated mice,* Cyp7α* mRNA increased approximately twofold and* Hmgr* decreased approximately threefold, probably as a feedback reaction to the elevated plasma cholesterol concentrations. Supplementation of BC to vitamin A-deficient diet significantly decreased* Cyp7α* mRNA levels possibly due to lower plasma cholesterol concentrations, while* Hmgr *mRNA levels were not affected.

## 4. Discussion

In this study, we showed that dietary vitamin A deficiency significantly accelerates atherogenesis in an atherosclerosis prone, apoE^−/−^ mouse model, despite unaffected plasma retinol and RA concentrations. We also found that a diet enriched with *β*-carotene can compensate for the effects of dietary vitamin A deficiency with regard to atherosclerosis development.

Analyzing the hepatic retinol reservoir, as an indicator for the available vitamin A in the animals, revealed a 30% hepatic total retinol pool after 15 weeks of vitamin A-deficient diet in comparison to mice fed a vitamin A-containing diet. This reduction is lower than the drop reported in other mouse strains fed with a vitamin A-deficient diet, SENCAR mice [30], aryl hydrocarbon receptor- (AHR-) null mice [31], and BALB/c mice [32]. The reason for the higher remaining pool in apoE^−/−^ mice is not known. As expected, decreased retinol concentrations were also detected in stellate cells isolated from the VAD group. As *β*-carotene is a source of vitamin A in most diets, including human diets [33], and *β*-carotene from* Dunaliella* was shown to be a precursor for retinol and retinyl esters in rats [34], we assumed that fortification of the vitamin A-deficient diet with *β*-carotene from* Dunaliella* would restore liver retinoids. Interestingly, fortification of the vitamin A-deficient diet with *β*-carotene isomers did not completely restore liver retinol and only a trend towards elevated total retinol concentrations was detected. Thus, liver retinol pool was comparable in the VAD and VAD-BC groups. We used Cyp26a1 to monitor liver RA concentrations [28] and as expected, VAD group had significantly lower mRNA levels of Cyp26a1, indicating lower levels of RA, and BC reverts this effect. The lack of effect of the treatments on plasma retinol or RA levels suggests dissociation between their plasma levels and liver concentrations at these diet regimes.

The two main isomers of *β*-carotene, all-*trans* and 9-*cis,* were accumulated in the liver as in rats fed* Dunaliella bardawil* powder [34] and in LDLR^−/−^ mice [6]. Lack of vitamin A in the diet caused significant hepatic elevation in 9-*cis* and all-*transβ*-carotene concentrations after *β*-carotene isomers supplementation compared to a vitamin A-containing diet enriched with these isomers. This may indicate that a lack of vitamin A in the diet and the ensuing liver reservoir decline resulted in better absorption and/or storage of *β*-carotene isomers. As retinoids are known to regulate the expression of carotenoid transporters [35], the higher concentrations of *β*-carotene isomers in the livers of the VAD group may be a result of higher expression of transporters involved in carotenoid absorption.

Plasma cholesterol concentrations were significantly increased in mice that were fed the vitamin A-deficient diet compared to the control group. These results contradict previous findings by Gatica et al., which showed no change in LDL and VLDL cholesterol concentrations in rats fed a vitamin A-deficient diet [19]. Similarly, Oliveros et al. demonstrated cholesterol and triglyceride concentration decrement in rats fed a vitamin A-deficient diet [20]. It is important to note that these experiments were conducted in animal models with a normal lipid profile, whereas we, for the first time, investigated the effects of vitamin A deficiency in a hyperlipidemic, atherosclerosis prone mouse model. The cholesterol elevation can be derived from increased synthesis, increased cholesterol absorption, or reduced plasma cholesterol clearance. In the analysis of* Hmgr* liver mRNA levels, we found a significant decrease in the VAD group. It seems that this decrease in liver* Hmgr* mRNA expression in the vitamin A-deficient diet was due to a feedback inhibition caused by the elevated plasma cholesterol concentrations in this group. A similar mechanism may have caused elevation in liver* Cyp7α* mRNA expression in the VAD group (the first enzyme in the cholesterol-bile acid pathway), probably in order to decrease cholesterol concentrations by turning it into bile acids. These results suggest that the increased concentrations of cholesterol are not due to increased cholesterol synthesis in the liver or decreased bile acid synthesis from cholesterol. The direct reason to the elevated plasma cholesterol in the VAD group is not known. However, the loss of retinol from HSC in the VAD group is an indication for stellate cell activation and development of liver inflammation [36]. As liver inflammation is associated with dyslipidemia [37] and has been suggested to contribute to atherosclerosis [38], we assume that it also affects cholesterol metabolism and contributed to atherogenesis in our mouse model.

The results clearly show that dietary vitamin A deficiency significantly increased the atherosclerotic lesion area, despite the lack of effects on plasma concentrations of retinol and all-*trans* RA. Studies on the effects of VAD on atherosclerosis have not been published and the only study investigating the effects on lipid accumulation in the blood vessels, by Gatica et al., demonstrated that the concentrations of triglycerides and total cholesterol increased in the aorta of rats treated with a vitamin A-deficient diet [39]. The results of the current study are in accordance with previous studies from our laboratory demonstrating that fortification of a diet containing vitamin A with the provitamin A carotenoids inhibits atherosclerosis in apoE^−/−^ and LDLR^−/−^ mouse models [5, 6].

It was unexpected to find that the remaining 30% liver retinoids reservoir did not prevent the increment in plasma cholesterol concentrations and accelerated atherogenesis and that dietary *β*-carotene can inhibit the accelerated atherogenesis without restoring the liver retinoid stockpile or increasing plasma retinoid concentrations. D'Ambrosio et al., in their review on vitamin A metabolism [40], highlighted the importance of postprandial retinyl ester uptake into extrahepatic tissues and suggested that this pathway can sufficiently provide tissue requirements. This opinion is supported by findings showing that humans who lack retinol binding protein (RBP4) [17] as well as RBP-deficient mice [18] are physiologically normal (RBP4-deficient mice). We suggest that dietary BC can supply vitamin A to peripheral tissue directly, as intact BC molecule, or by its conversion to retinyl ester in the intestine or in peripheral tissues expressing BCMO1. In a recent study we showed that BCMO1 is expressed and is active in macrophages (accepted PLOS1) and previous studies demonstrated its expression in adipocytes [41]. Hence, delivery of BC and its conversion to retinoids in BCMO1-expressing cells in peripheral tissues can potentially retard atherosclerosis without restoring liver retinol pools.

In summary, using an atherosclerotic mouse model, we demonstrated that a diet low in vitamin A can be indicated as a potential risk factor for atherosclerosis. Moreover, we suggest that dietary carotenoids as a sole source for vitamin A can compensate for vitamin A deficiency.

## Abbreviations

apoE^−/−^: Apolipoprotein E deficient
BC: * Dunaliella* treated group
BCMO1: *β*-carotene 15,15′ monooxygenase 1
HSC: Hepatic stellate cell
RA: Retinoic acid
VAD: Vitamin A-deficient diet group
VAD-BC: Vitamin A-deficient diet fortified with* Dunaliella* group.
